# Arterial stiffness in patients with coronary artery disease: relation with in-stent restenosis following percutaneous coronary intervention

**DOI:** 10.1186/s12872-016-0305-4

**Published:** 2016-06-06

**Authors:** Zrinko Prskalo, Ivica Brizić, Darko Markota, Ivica Markota, Mladen Boban, Monika Tomic, Boris Starcevic

**Affiliations:** Department of Cardiology, University Hospital Mostar, Bijeli Brijeg bb, 88 000 Mostar, Bosnia and Herzegovina; Department of Nephrology, University Hospital Mostar, Mostar, Bosnia and Herzegovina; Department of Pharmacology, University of Split, Split, Croatia; Department of Cardiology, University Hospital Dubrava, Zagreb, Croatia

**Keywords:** Vascular stiffness, Coronary artery disease, Coronary restenosis

## Abstract

**Background:**

Coronary artery disease (CAD) is one of the most important issues in modern medicine due to its high mortality and prevalence. An early detection and prevention can reduce morbidity and mortality. Arterial stiffness is a potent and independent predictor of CAD. We aimed to investigate the arterial stiffness in CAD patients undergoing the coronary angiography. Also, we investigated a possible correlation between arterial stiffness and in-stent restenosis (ISR), an important limitation of percutaneous coronary intervention (PCI).

**Methods:**

The study included 160 patients undergoing coronary angiography, treated either with PCI or with CABG. The pulse wave velocity (PWV) and augmentation index (AIx) were measured by the Arteriograph.

**Results:**

PWV in the CAD group (12.24 ± 2.78 m/s) was significantly higher compared to the control group (8.27 ± 1.89 m/s). PWV in ISR and left main (LM) stenosis group (14.03 ± 3.15 and 13.89 ± 2.95 m/s) was significantly higher compared to the control and CAD groups. Peripheral and central AIx were significantly higher in CAD group (1.38 ± 30.63 % and 38.35 ± 15.52 %) than in control group (−11.35 ± 26.74 % and 26.91 ± 10.62 %). Patients with LM stenosis have significantly higher values of peripheral and central AIx (23.37 ± 23.77 % and 49.71 ± 12.02 %) than the CAD and ISR group.

**Conclusions:**

The study confirmed a positive correlation between arterial stiffness measures, PWV and AIx, and CAD. Also, this study showed the correlation between PWV and ISR which can help to select more appropriate stent.

**Electronic supplementary material:**

The online version of this article (doi:10.1186/s12872-016-0305-4) contains supplementary material, which is available to authorized users.

## Background

Coronary artery disease (CAD) is one of the most important issues in modern medicine due to high prevalence and mortality. Both, the prevention and treatment of CAD in recent decades have been improved, however, CAD remains the first inductor of morbidity and mortality in humans [1]. An early detection and prevention of CAD is very important and can reduce morbidity and mortality. Numerous epidemiological and clinical studies showed a positive correlation between CAD and male gender, high blood pressure, diabetes, smoking, physical inactivity, and hyperlipidemia [2]. In addition to these traditional risk factors, the arterial stiffness defined as a direct measure - aortic pulse wave velocity (PWV) and an indirect parameter - augmentation index (AIx), is a strong and independent predictor of CAD and adverse cardiovascular events [3, 4]. The majority of the current research, investigating correlation between arterial stiffness and CAD was of observational or epidemiological nature [5, 6]. Only few clinical studies showed a direct correlation between PWV and CAD diagnosed by coronary angiography [7, 8].

Treatment of CAD could involve pharmacotherapy, percutaneous coronary interventions (PCI), and surgical revascularization. All methods are associated with some limitations and disadvantages. In the past few decades the development of PCI, atherectomy, with bare-metal (BMS) and drug eluting stents (DES) has made a significant progress in the management of CAD [1]. The most important limitation of PCI is thrombosis and in-stent restenosis (ISR). The incidence of ISR after a BMS implantation is 20–25 % in the first 6 months [9]. The development of DES contributed to a significant reduction of the ISR, as it decreased the incidence rate of ISR in only 5–10 % cases [10–12]. However, the rate of DES use is lower than 10 % in most countries due to the high cost. The incidence of DES implantation in developing countries, such as ours, is smaller than an average rate, because of its higher cost.

Factors that contribute to ISR are not fully understood. Cassese et al. showed that diabetes mellitus, multiple lesions, small vessels, multiple stents, smaller final stent lumen diameter and stent design are predictors for ISR [11, 13]. Some previous studies established a link between arterial stiffness and ISR. Ueda et al. found a correlation between AIx in the ascending aorta and ISR [14]. Also, Nakayama et al. showed a correlation between pulse pressure and ISR [15]. Recently, Mahfouz et al. described a correlation between arterial stiffness measured with ultrasonography and ISR [16].

In this study we aimed to investigate the correlation between CAD assessed by coronary angiography graded by SYNTAX score and arterial stiffness measured by Arteriograph, a simple, inexpensive, reproducible and investigator-independent method. Also, we investigated the correlation between ISR and aortic PWV and Aix.

## Methods

The study included 160 patients with CAD who underwent an elective coronary angiography in the Department of Invasive Cardiology of Clinical Hospital Mostar from 1^st^ April 2014 to 1^st^ October 2014. The control group consisted of 59 sex and age-matched healthy people. All subjects in the control group underwent the clinical examination, ECG, stress test, echocardiography, measuring of arterial stiffness and laboratory testing. All those subjects had a clear medical history. The CAD group excluded subjects with atrial fibrillation, acute coronary syndrome, and with significant valvular disease. All CAD patients had previous concordant non-invasive findings for CAD and experienced angina pectoris. They received an appropriate treatment (statines, beta blockers, ACE inhibitors and aspirin) in accordance with the guidelines for CAD [17]. Out of a total number of CAD patients, 102 were treated with PCI and 58 underwent heart surgery. During the next 6 months 23 patients had ISR in BMS. All patients who underwent PCI had dual antiplatelet therapy, aspirin and clopidogrel. The study was conducted in accordance with the Helsinki Declaration and approved by the Ethics Committee of the University Hospital Mostar. All subjects gave written informed consent to participate in this study.

### Coronary angiography and SYNTAX score

All patients underwent routine coronary angiography using the Judkins technique on digitized coronary angiography equipment (Shimadzu, Kyoto, Japan). Coronary angiograms were computerized and assessed by two experienced angiographers who were blinded to the results of arterial stiffness measurements. A significant CAD was defined as at least 50 % or more stenosis. ISR was defined as >50 % diameter stenosis at the stent site.

The SYNTX score is a semi-quantitative angiographic tool to determine the extent of CAD [18]. The algorithm contains of 12 questions referring to the coronary anatomy and total number and extent of coronary artery lesions. The SYNTAX score was calculated for each coronary lesion producing a ≥50 % luminal obstruction in vessels with a diameter of 1.5 mm or more. Patients were divided according to 2-year rates of major adverse coronary events as low (0–22), intermediate (23–32), and high (≥33) risk group. The SYNTAX score was calculated with a computer-based questionnaire program.

### Measurements of arterial stiffness

Arterial stiffness was measured by an oscillometric non-invasive device Arteriograph (TensioMed, Budapest, Hungary). The device detects and processes oscillations on the upper-arm positioned cuff by a special high fidelity sensor during a complete occlusion of brachial artery. Simultaneously with the parameters of arterial stiffness (PWV (m/s) an AIx (%), the device also recorded systolic and diastolic blood pressures and heart rate [19].

Twenty-four hours prior to the examination subjects were asked to refrain from exercise, fruits, vegetables, dietary supplements, tea, alcoholic beverages, and caffeine containing foods. Also, 12 hours before measurement of arterial stiffness patients did not take any drugs. All experiments were carried out in a quiet, temperature controlled room maintained around 24 °C and were started at 8 a.m. The subjects had rested quietly for 15 min in the supine position before the measurement.

### Statistics

Data are expressed as mean ± SD. Statistical analyses were performed using GraphPad Instat and GraphPad Prism (San Diego, CA USA). All variables were normalized before data statistical analyses on age, heart rate and blood pressure. One-way ANOVA test was used to evaluate changes in AIx and PWV. When statistical significance was reached by ANOVA (*P* <0.05), Bonferroni test was used for the post hoc analysis.

## Results

In this study we investigated the association of arterial stiffness with CAD and correlation between degrees of CAD measured by SYNTAX score. We found a strong correlation between CAD and arterial stiffness, but no association between degrees of the CAD with the parameters of arterial stiffness. From CAD group we extracted CABG, PCI, left main (LM) stenosis and ISR groups.

The general characteristics of control, CAD, ISR and LM stenosis groups are shown in Table 1. In the control and all experimental groups the male sex was dominant. Also, in the CAD, ISR and LM stenosis group, patients were slightly older with higher mean arterial pressure.

**Table 1:** General characteristics of control, CAD and ISR groups

	Control group (*n*-58)	CAD group (*n*-160)	ISR group (*n*-23)	LM stenosis group (*n*-9)
Age (year)	59.5 ± 5.4	61.5 ± 4.8	62.1 ± 6.1	63.8 ± 8.4
Male *n* (%)	43 (72.9)	115 (71.7)	22 (95.7)	9 (100)
Female *n* (%)	16 (27.1)	45 (28.3)	1 (4.3)	0 (0)
Smoking *n* (%)	21 (35.6)	82 (51.3)	8 (34.8)	6 (66.6)
Cholesterol (mmol/L)	5.59 ± 1.45	5.38 ± 1.33	5.06 ± 1.56	5.45 ± 1.73
LDL (mmol/L)	3.84 ± 0.78	3.66 ± 0.51	3.58 ± 0.68	3.78 ± 1.44
HDL (mmol/L)	1.02 ± 0.43	1.08 ± 0.25	1.13 ± 0.58	1.06 ± 0.29
Triglycerides (mmol/L)	2.12 ± 0.91	1.92 ± 0.77	1.84 ± 0.98	1.79 ± 0.43
Heart rate (beats/min)	65.7 ± 6.1	64.1 ± 4,2	65.1 ± 9,7	64.7 ± 8,9
Mean blood pressure (mm Hg)	101.3 ± 5.6	104.6 ± 6,5	105.7 ± 8.9	106.1 ± 9.6
Diabetes mellitus *n* (%)	0.0 (0.0)	25 (15.7)	3 (13.0)	0 (0)
Glucose blood level (mmol/L)	5.17 ± 0.67	5.71 ± 1.73	8.23 ± 0.72	5.34 ± 0.89
Renal failure *n* (%)	0.0 (0.0)	6 (3.75)	1 (4.3)	1 (11,1)
Creatinine (μmol/L)	94.22 ± 22.96	98.30 ± 26.04	143	109.78 ± 32.12
SYNTAX scores		25.27 ± 4.1	19.33 ± 6.9	36.78 ± 14.47
Multiple stents *n* (%)		14 (16.1)	4 (17.3)	
Stent diameter (mm)		3.01 ± 0.78	3.09 ± 0.93	
Length of stent (mm)		18.4 ± 0.58	18.1 ± 2.42	

Data are shown as mean value ± SD

*CAD* coronary artery disease, *HDL* low-density lipoprotein, *ISR* in-stent restenosis, *LM* left main, *LDL* high-density lipoprotein, *SD* standard deviation

**Fig. 1:**
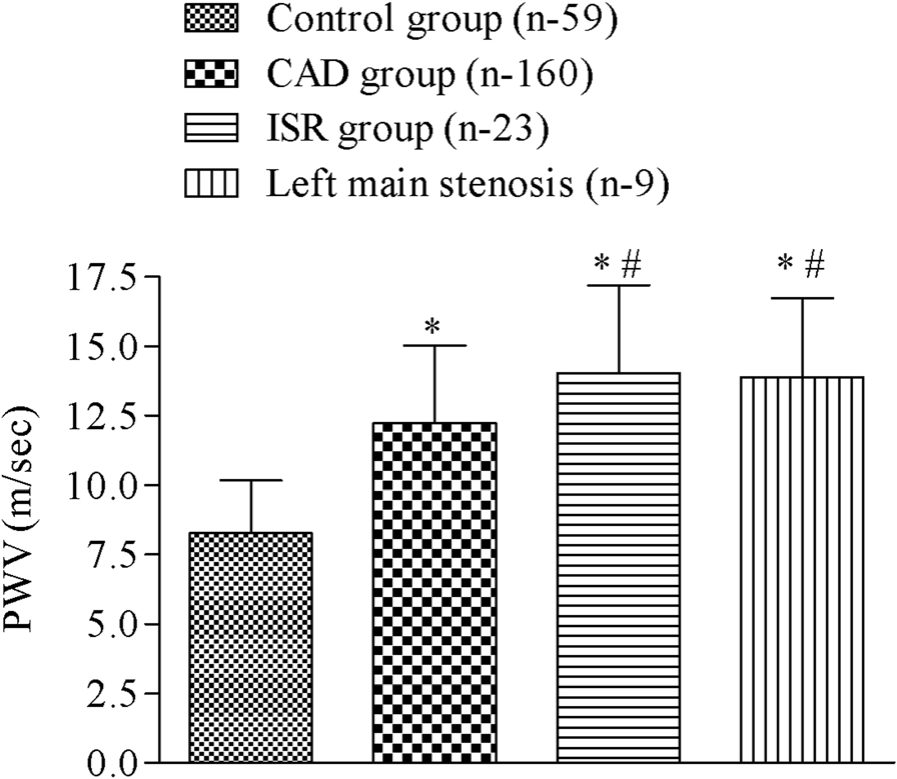
The values of aortic pulse wave velocity (PWV) in the control, coronary artery disease (CAD), in-stent restenosis (ISR) and left main stenosis group. Data are shown as mean ± SD, * *p* <0.05 vs control group, # *p* <0.05 vs CAD group

Aortic PWV in the CAD group (12.24 ± 2.78 m/s) was significantly higher compared to the control group (8.27 ± 1.89 m/s) (*P* <0.05). Also, PWV of an ISR and LM stenosis group (14.03 ± 3.15 and 13.89 ± 2.95 m/s) was significantly higher than in the control and CAD group (*P* <0.05) (Fig. 1).

**Fig. 2:**
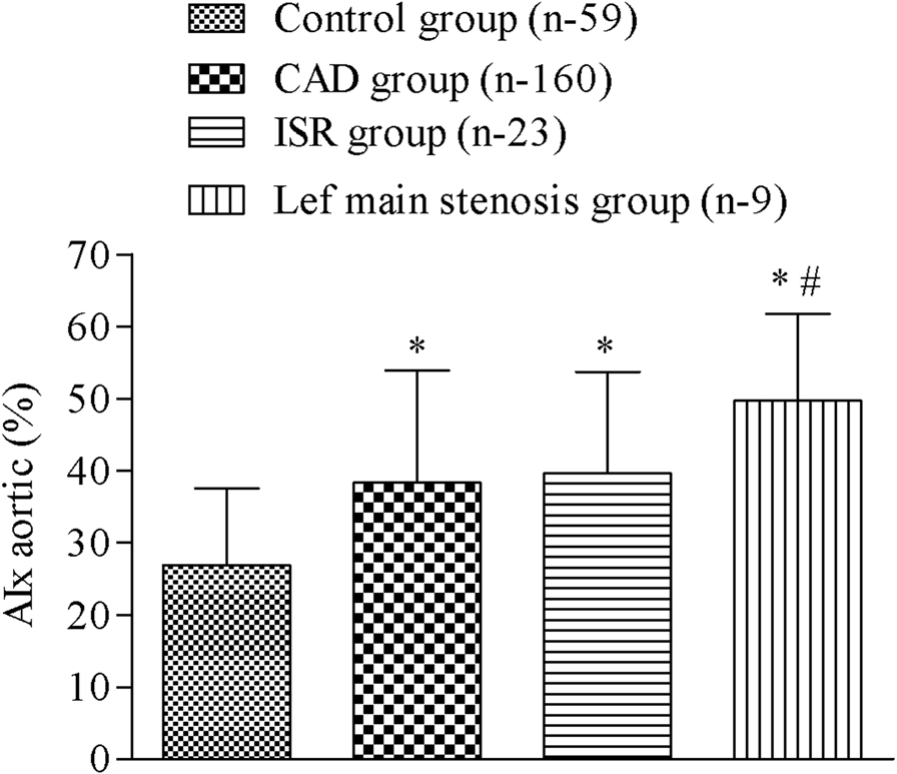
The values of aortic augmentation index (AIx), expressed in percentages in the control, coronary artery disease (CAD), in stent restenosis (ISR) and left main stenosis group. Data are shown as mean ± SD, * *p* <0.05 vs control group, # *p* <0.05 vs CAD and ISR groups

The central AIx in the CAD, ISR and LM stenosis group (38.35 ± 15.52, 39.71 ± 13.97 and 49.71 ± 12.02 %, respectively) was significantly higher compared to the control group (26.91 ± 10.62 %) (*P* <0.05). Also, central AIx of an LM stenosis group was significantly higher than in the CAD and ISR group (*P* <0.05) (Fig. 2).

**Fig. 3:**
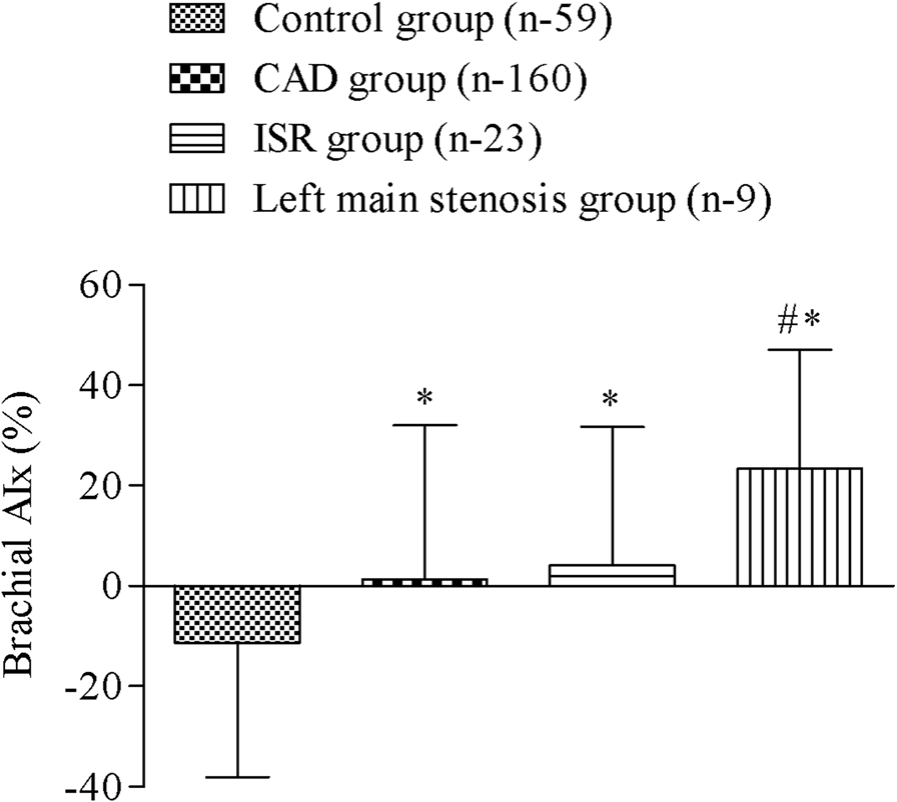
The difference between the brachial augmentation index (AIx) in control, coronary artery disease (CAD), in stent restenosis (ISR) and left main stenosis group. Data are shown as mean ± SD, * *p* <0.05 vs control group, # *p* <0.05 vs CAD and ISR groups

The peripheral AIx in the CAD, ISR and LM stenosis group (1.38 ± 30.63 and 4.09 ± 27.60 and 23.37 ± 23.77 %, respectively) was significantly higher compared to the control group (−11.35 ± 26.74 %) (*P* <0.05). Also, peripheral AIx of an LM stenosis group was significantly higher than in the CAD and ISR group (*P* <0.05) (Fig. 3).

The SYNTAX score was significantly higher in patients who were referred for CABG and patients with LM stenosis (34.62 ± 12.46 and 36.78 ± 16.47) compared to the PCI and ISR group (17.63 ± 9.77 and 19.33 ± 6.9) (*P* <0.05). Among patients treated with PCI and patients who underwent CABG we did not find a difference in the parameters of arterial stiffness (CABG *vs* PCI group; PWV 12.23 ± 2.93 *vs* 12.26 ± 2.68 m/s; central Aix 37.93 ± 14.36 *vs* 38.47 ± 15.39 %; peripheral AIx 1.35 ± 28.32 *vs* 1.57 ± 30.38 %) (Additional file 1).

## Discussion

In this study we showed a positive correlation between arterial stiffness parameters and CAD proved with coronary angiography. Also, our study showed the correlation between PWV and ISR.

Some previous studies showed the correlation between arterial stiffness and CAD [20–22]. In the most of them CAD was proved with non-invasive methods. Several studies demonstrated the correlation between CAD examined with coronary angiography and arterial stiffness. Liu et al. showed positive correlation between arterial stiffness measured by Sphygmocor and CAD proved with multi slice computed tomography coronary angiography [23]. Imanishi et al. concluded that high brachial–ankle PWV is an independent predictor for the presence of CAD, especially in men [24]. Contrary to our investigation, the arterial stiffness in the mentioned studies was proved with different devices and at different blood vessels or coronary artery disease was not identified at coronary angiography. Only one study investigated the correlation between CAD and PWV measured by Arteriograph. Similar to our study, they found the positive correlation between CAD examined with coronary angiography and arterial stiffness [7]. However, despite high differences between PWV and AIx in the control and CAD group, we also did not find the correlation between the CAD grades (defined by SYNTAX score) with PWV and AIx. But still, in this study we proved a correlation between arterial stiffness and left main stenosis. However, Cho et al. showed the correlation between AIx and the grade of CAD in patients aged less than 65 years, but not in the older ones [25]. The correlation was not found in patients above 65 years. In our opinion, it is not realistic to expect that even this method can determine the level of CAD. Also, the measuring of arterial elasticity is developed in order to asses an increased cardiovascular risk but some other specific methods should be used to confirm and prove CAD.

Furthermore, we investigated a correlation between ISR and arterial stiffness. The correlation between PWV and ISR was found, but not between AIx and ISR. Arterial stiffness is strongly dependent on the balance of two major proteins, elastin and collagen [26]. Normally, there is a tightly regulated balance between synthesis and degradation of these two proteins. Therefore, if there is an increased collagen production and reduced degradation in the whole vascular system, arterial stiffness will be increased and more likely an ISR will occur [27]. After the stent insertion some inflammatory changes in the coronary artery wall such as endothelial degradation, macrophage infiltration and smooth muscle cell proliferation could occur. Also, the inflammation in arteries increases the extracellular matrix proteins and collagen synthesis contributing to the ISR [28, 29]. BMS causes neointimal hyperplasia after implantation and leads to ISR and reintervention in more than 20 % of patients by 6 months [10, 11]. Similarly to our study, Ueda et al. showed the correlation between aortic stiffness and restenosis after balloon angioplasty [30]. However, mechanisms including collagen turnover in ISR and restenosis after balloon angioplasty are different. Stenting causes an even greater increase in collagen accumulation compared with balloon angioplasty [28, 31, 32]. Accordingly, the same author showed the correlation between aortic AIx and ISR [14], although the correlation between Aix and ISR was not found in our study. Recently, Mahfouz et al. proved the association of ISR and arterial elasticity [16]. All studies that have found a correlation between the extent of ISR and arterial stiffness were performed with various devices and methods. All results can contribute to a better understanding of ISR and help to make a decision considering stent implantation. In the cases of high PWV and other risks factors for ISR a cardiologist may be guided to implant DES. Moreover, this could be very useful in a cath lab with limited finances.

## Conclusion

Our results confirm the importance of measuring arterial elasticity in patients with CAD predisposition. Also, we have found that an elevated PWV is associated with ISR suggesting its measuring before the procedure which contributes for a better selection of the stent implant. This study confirms the value of Arteriograph in cardiovascular risk assessment as a broadly applicable method for screening the general population.

## Abbreviations

ACE, angiotensin-converting-enzyme; AIx, augmentation index; BMS, bare-metal stent; CABG, coronary artery bypass graft; CAD, coronary artery disease; DES, drug eluting stent; ISR, in-stent restenosis; LAD, left anterior descending artery; LM, left main; PCI, percutaneous coronary intervention; PWV, pulse wave velocity

## Additional file

Additional file 1: The study data. (XLSX 16 kb)
